# A bioassay for the detection of benzimidazoles reveals their presence in a range of environmental samples

**DOI:** 10.3389/fmicb.2014.00592

**Published:** 2014-11-13

**Authors:** Terence S. Crofts, Yujie Men, Lisa Alvarez-Cohen, Michiko E. Taga

**Affiliations:** ^1^Department of Plant and Microbial Biology, University of California at BerkeleyBerkeley, CA, USA; ^2^Department of Civil and Environmental Engineering, University of California at BerkeleyBerkeley, CA, USA

**Keywords:** 5, 6-dimethylbenzimidazole, benzimidazole, *S. meliloti*, bioassay, environment, cobamide, vitamin B_12_

## Abstract

Cobamides are a family of enzyme cofactors that include vitamin B_12_ (cobalamin) and are produced solely by prokaryotes. Structural variability in the lower axial ligand has been observed in cobamides produced by diverse organisms. Of the three classes of lower ligands, the benzimidazoles are uniquely found in cobamides, whereas the purine and phenolic bases have additional biological functions. Many organisms acquire cobamides by salvaging and remodeling cobamides or their precursors from the environment. These processes require free benzimidazoles for incorporation as lower ligands, though the presence of benzimidazoles in the environment has not been previously investigated. Here, we report a new purification method and bioassay to measure the total free benzimidazole content of samples from microbial communities and laboratory media components. The bioassay relies on the “calcofluor-bright” phenotype of a *bluB* mutant of the model cobalamin-producing bacterium *Sinorhizobium meliloti*. The concentrations of individual benzimidazoles in these samples were measured by liquid chromatography-tandem mass spectrometry. Several benzimidazoles were detected in subpicomolar to subnanomolar concentrations in host-associated and environmental samples. In addition, benzimidazoles were found to be common contaminants of laboratory media components. These results suggest that benzimidazoles present in the environment and in laboratory media have the potential to influence microbial metabolic activities.

## INTRODUCTION

**FIGURE 1:**
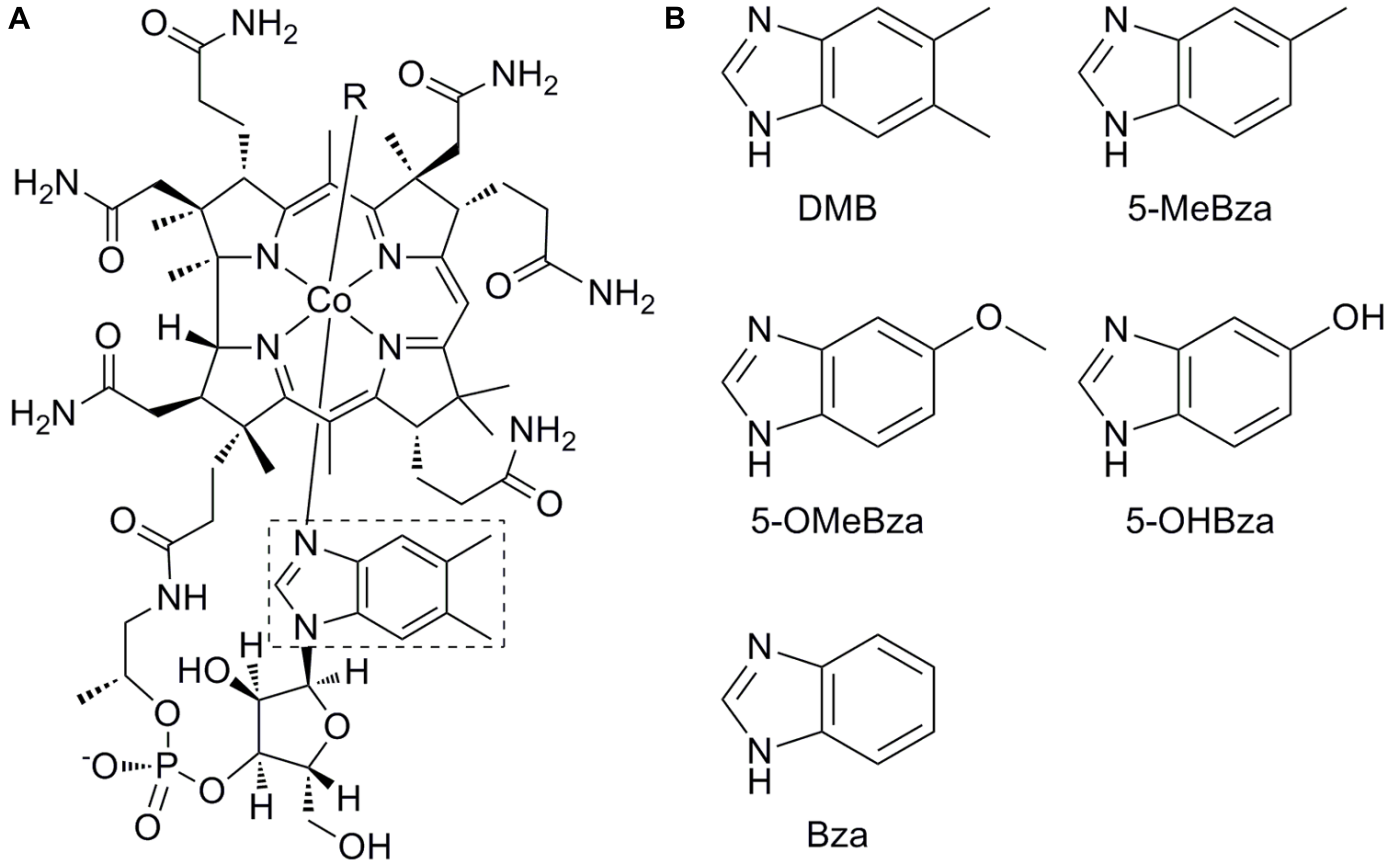
**Structure of cobalamin and benzimidazole lower ligands. (A)** Structure of cobalamin. The lower ligand, 5,6-dimethylbenzimidazole (DMB) is boxed. **(B)** Structures of lower ligands used in this study. 5-methylbenzimidazole (5-MeBza), 5-methoxybenzimidazole (5-OMeBza), 5-hydroxybenzimidazole (5-OHBza), and benzimidazole (Bza).

Cobamides are cofactors that function in a variety of metabolic processes in animals, protists, and prokaryotes. Cobamides belong to a broader class of molecules called corrinoids that share a common cobalt-containing corrin ring. Cobamides are corrinoids that contain a lower axial ligand covalently bound to the corrin ring via the nucleotide loop (Roth et al., 1996; Escalante-Semerena, 2007). The essential nutrient cobalamin (**Figure 1A**, known as vitamin B_12_ when taken in its cyanated form as a supplement) is the best studied cobamide and is well known for its importance in human health. Cobalamin and other cobamides also function as cofactors for several ecologically important processes in prokaryotes. For example, cobamides are used as cofactors for methanogenesis, acetogenesis, carbon fixation, the formation of toxic methyl mercury, and the detoxification of chlorinated solvents such as trichloroethene (TCE; Choi et al., 1994; Banerjee and Ragsdale, 2003; He et al., 2007; Ragsdale and Pierce, 2008). Cobamides are found in host-associated and environmental microbial communities including the human intestine, bovine rumen, wood-feeding insects, and TCE-dechlorinating communities (Kräutler et al., 2003; Allen and Stabler, 2008; Girard et al., 2009; Yi et al., 2012).

The upper (β) ligand of cobamides is the catalytic site (labeled R in **Figure 1A**). Adenosylcobamides (*R* = 5′-deoxyadenosine) catalyze rearrangement reactions via a radical intermediate, while methylcobamides (*R* = CH_3_) catalyze methyltransfer reactions (Banerjee and Ragsdale, 2003). The cobamide lower (α) ligand is the main site of structural diversity, though the biological role for this diversity is unclear. Lower ligands of cobamides include benzimidazoles such as 5,6-dimethylbenzimidazole (DMB), the lower ligand of cobalamin (**Figure 1**); purines; and phenolics (Renz, 1999). Of these classes, the purines and phenolics have other biological functions, while the benzimidazoles are thought to function exclusively as cobamide lower ligands.

There is growing evidence that cobamides and their biosynthetic precursors are shared among microbes in complex communities (Seth and Taga, 2014). Bioinformatic analyses indicate that the majority of bacteria that are capable of using cobamides do not synthesize them *de novo*, but instead import cobamides produced by other organisms (Zhang et al., 2009; Degnan et al., 2014). Cobamide biosynthetic intermediates such as cobinamide (Cbi) and α-ribazole are also taken up and used by organisms that lack the enzymes for their biosynthesis (Gray and Escalante-Semerena, 2010; Yi et al., 2012). Additionally, many prokaryotes including *Escherichia coli* lack the complete cobamide biosynthetic pathway but can attach a lower ligand to a cobamide precursor (corrinoid salvaging), and some, such as *Dehalococcoides mccartyi*, can remove and replace the lower ligand to form a different cobamide (cobamide remodeling; Woodson and Escalante-Semerena, 2004; Escalante-Semerena, 2007; Gray and Escalante-Semerena, 2009; Yi et al., 2012). Some prokaryotes capable of synthesizing cobamides *de novo* can also attach an exogenously provided lower ligand to form a different cobamide, a process known as guided biosynthesis (Perlman, 1959, 1971). Corrinoid salvaging, cobamide remodeling, and guided biosynthesis activities require the presence of a free lower ligand base such as DMB in the environment (Escalante-Semerena, 2007). Indeed, the availability of DMB may be crucial for the production of biologically active cobamides by environmental bacteria such as *D. mccartyi* (Men et al., 2014b). Interestingly, although the availability of free lower ligands in microbial communities has not been investigated previously, DMB has been reported to be a contaminant of common laboratory agar (Anderson et al., 2008).

Here, we have developed a new method for the purification, detection, and quantification of free benzimidazoles that are commonly found as cobamide lower ligands. We report the development of a bioassay for the quantification of benzimidazoles based on the “calcofluor-bright” (CFB) phenotype of *Sinorhizobium meliloti* mutants that lack a functional *bluB* gene, which encodes the enzyme responsible for the oxygen-dependent biosynthesis of DMB (Campbell et al., 2006; Taga et al., 2007). The CFB phenotype is indicative of an increased abundance or structural alteration in the exopolysaccharide (EPS) succinoglycan, and this phenotype has been observed previously in strains containing mutations in genes involved in amino acid starvation and the regulation, biosynthesis, and processing of succinoglycan (Finan et al., 1985; Doherty et al., 1988; Wells and Long, 2002; Campbell et al., 2006; Gibson et al., 2006; Taga et al., 2007; Pinedo et al., 2008; Morris and González, 2009). In this work we have found that the CFB phenotype of the *bluB* mutant results from a deficiency in the activity of the cobalamin-dependent ribonucleotide reductase (RNR) enzyme and is due to a previously unrecognized link between the CFB phenotype and DNA stress. By developing and applying a bioassay based on the CFB phenotype, we show that benzimidazoles are present in subpicomolar to subnanomolar concentrations in a variety of environmental samples, as well as in common components of laboratory media.

## MATERIALS AND METHODS

### BACTERIAL STRAINS AND CULTURE CONDITIONS

All *S. meliloti* strains are derivatives of the wild type (WT) strain Rm1021 (Meade et al., 1982). The *bluB::gus* Gm^R^ strain was used for the CF bioassay (Campbell et al., 2006). *S. meliloti* cultures were grown with aeration at 30°C in LB medium supplemented with 2.5 mM CaCl_2_ and 2.5 mM MgSO_4_ (LBMC), or in M9 minimal medium with 0.2% sucrose, 10 μg/L biotin, 1 mg/mL methionine, and 10 μM CoCl_2_ (Maniatis et al., 1982). For growth on solid media, technical grade agar (Difco) was added at a final concentration of 15 mg/mL. When necessary, antibiotics were added at the following concentrations (μg/mL): gentamicin, 25; tetracycline, 10; streptomycin, 500; and spectinomycin, 100.

### *S. meliloti* CALCOFLUOR PHENOTYPE ON AGAR PLATES

To assay the CF phenotype on solid media, 100 μL of a liquid culture was spread on LBMC agar containing 0.02% CF and 10 mM HEPES buffer at pH 7.4. Sterile filter disks were placed on the agar surface, and the compound being tested was applied to each disk as indicated. The plates were incubated for 2–3 d at 30°C and then photographed under ultraviolet (UV) light. 10 μL of each tested compound was added, except when indicated, at the following concentrations: nalidixic acid, 50 mM; mitomycin C, 2 mg/mL (30 μL); cisplatin, 0.8 mg/mL (20 μL); methyl methanesulfonate (MMS), 1%; 4-Nitroquinoline-1-Oxide (4NQO), 2 mg/mL (30 μL); gentamicin, 50 mg/mL; neomycin, 200 mg/mL; chloramphenicol, 20 mg/mL; tetracycline, 0.1 mg/mL; methanol, 95% (20 μL); *N*-butanol, 99%; cobalt chloride, 1 M; iron(III) chloride, 1 M; Ethylenediaminetetraacetic acid (EDTA), 0.5 M; ethylene glycol tetraacetic acid (EGTA), 0.5 M; sodium hydroxide (NaOH), 5 N; formic acid, 80%, Tween, 99%, Triton X-100, 99%, dimethyl sulfoxide (DMSO), 99%, hydroxyurea, 100 mM; hydrogen peroxide, 10%; menadione, 9 mg/mL; cumene hydroperoxide, 8%; and sodium dodecyl sulfate (SDS), 10%.

### *S. meliloti* CALCOFLUOR BIOASSAY TO DETECT BENZIMIDAZOLES

Three independent cultures of *S. meliloti bluB::gus* Gm^R^ were grown in LBMC liquid medium for 40–48 h. The cultures were diluted in M9 to an O.D._600_ of 0.04 in 96-well microtiter plates and mixed with known concentrations of DMB standards or dilutions of a sample to be tested, in a total volume of 200 μL per well. All solutions were sterilized by passing through a 0.22 μm or 10,000 molecular weight cut-off filter (MWCO, Pall, Port Washington, New York, USA) prior to use. The edges of the plates were sealed with parafilm and the plates were placed on a moist paper towel in a sealed plastic bag to limit evaporation. The plates were incubated in a shaking incubator at 30 °C for 55–70 h. CF was added to a final concentration of 80 μg/mL, bringing the total volume to 300 μL per well, and the plate was incubated at room temperature in the dark for an additional 5 h. Fluorescence was measured using a BioTek Synergy 2 plate reader with excitation at 360 nm and emission measured at 460 nm. Sigmoidal curves were solved using the KaleidaGraph program with the equation *y* = m1 + (m2–m1)/(1 + (x/m3)ˆm4), where *y*, m1, m2, m3, m4, and *x* correspond to the observed fluorescence at a given dilution factor, the minimum fluorescence value, the maximum fluorescence value, the EC_50_, the slope at EC_50_, and the dilution factor of the sample, respectively. The EC_50_ error was calculated by the program.

### SAMPLE PREPARATION

5-hydroxybenzimidazole (5-OHBza) was synthesized and purified from 5-methoxybenzimidazole (5-OMeBza) as previously described (Renz et al., 1993; Crofts et al., 2013). DMB (Sigma–Aldrich), 5-methylbenzimidazole (5-MeBza, Acros Organics), 5-OMeBza (Sigma–Aldrich), and benzimidazole (Bza, Sigma–Aldrich) were purchased in powder form and stored at -20°C as DMSO stock solutions.

To obtain cell-free supernatants, cultures of WT *S. meliloti* Rm1021 and *bluB* mutant strains containing the plasmids pMS03-*bluB* (Yu et al., 2012) or pJL1031 (*bluB* cloned under its native promoter in pFAJ1700; Campbell et al., 2006) were grown in M9 liquid medium for 48 h. Samples were diluted in M9 to an O.D._600_ of 0.75 and supernatants were collected by centrifugation at 9,000 g for 5 min. Sample were passed through a 10,000 MWCO filter to remove cells.

Seven and half gram of technical grade agar (Difco), plant cell culture tested agar (Sigma), noble agar (US Biological), agarose (Fisher), and yeast extract (Becton Dickinson) were suspended in 300 mL of phosphate buffered saline (PBS) adjusted to pH 8.0. Rumen fluid from two fistulated cows fed on a high forage diet (Cows 1921 and 1927, a gift from Ed DePeters, UC Davis Department of Animal Science) was collected in September, 2009, and stored at -20°C. Thirty-five milliliter aliquots of rumen fluid were clarified by centrifugation and diluted to 75 mL in PBS adjusted to pH 8.0.

Ten gram of termite fecal pellets obtained from a laboratory colony of *Zootermopsis* sp. was suspended in 75 mL of PBS and the pH was adjusted to 8.0. Topsoil from the UC Berkeley campus Eucalyptus Grove and soil adjacent to Strawberry Creek on the UC Berkeley campus (GPS coordinates 37.870958, -122.264433) were collected in September, 2013. Ten gram of each sample was suspended in 75 mL of PBS and the pH was adjusted to 8.0. These samples were clarified by passing through a syringe containing a cotton plug. Water from Strawberry Creek was collected from an adjacent location. A 6.5 by 45 cm Winogradsky column (a gift from Sydney Kustu) was established over 20 years ago with San Francisco bay mud and maintained in the lab with natural sunlight and periodically refilled with settled deionized water. Aqueous samples (1 L of creek water or 75 mL of the upper portion of the Winogradsky column) were mixed with NaCl, KCl, Na_2_HPO_4_, and K_2_HPO_4_ to adjust the salt and buffer content to that of PBS, and the pH was subsequently adjusted to 8.0.

### PURIFICATION OF BENZIMIDAZOLES

Samples were mixed with an equal volume of ethyl acetate (ACS certified grade) and shaken vigorously in a separatory funnel. The organic layer was removed, and the extraction of the aqueous layer was repeated with an additional volume of ethyl acetate. The organic phase from the two extractions were pooled, and solvent was removed by rotary evaporation at 50°C. Samples were resuspended in PBS and applied to a 360 mg C18 Sep-Pak (Waters, Milford, Massachusetts) previously activated with 3 mL of methanol and equilibrated with 6 mL deionized water. The cartridge was washed with 6 mL of 30% methanol and eluted with 2 mL of 70% methanol. The eluate was collected and dried under reduced pressure at 45°C. The final product was resuspended in deionized water, filtered through a 10,000 MWCO spin filter, and stored at -20°C prior to analysis.

### HIGH PERFORMANCE LIQUID CHROMATOGRAPHY (HPLC) AND LC/MS/MS ANALYSIS

To determine the efficiency of the extraction method, 250 nmol of DMB, 5-MeBza, 5-OMeBza, 5-OHBza, benzimidazole (Bza), and cobalamin were extracted as described above. Samples were analyzed with an Agilent 1200 series high performance liquid chromatography (HPLC) system equipped with a diode array detector. Samples were analyzed on an Agilent SB-Aq 4.5 × 150 mm column (5 μm pore size) at 1.5 mL/min at 40°C with a gradient of 2–100% buffer B over 10 min, where buffer A was 10 mM ammonium acetate pH 6.5 and buffer B was 100% methanol. Quantification was achieved by comparing peak areas at 280 nm with standards of known concentration. Liquid chromatography-tandem mass spectrometry (LC/MS/MS) was carried out on an Agilent 6410 liquid chromatograph-triple quadrupole mass spectrometer with multiple reaction monitoring, using a recently reported quantitative method (Men et al., 2014b). Briefly, samples were analyzed on a 3.0 × 50 mm Agilent C18 Eclipse Plus column (1.8 μm pore size) at 0.5 mL/min at 40°C with a gradient of 18–21% buffer B over 3 min and 21% buffer B for 2 min, where buffer A was 0.1% formic acid in water and buffer B was 0.1% formic acid in methanol. The following precursor and product ions were monitored: m/z119.1 and 65 for Bza, m/z 133.1 and 77 for 5-MeBza, m/z 147.1 and 131 for DMB, m/z 149.2 and 79 for 5-OMeBza, and m/z 135.1 and 53 for 5-OHBza. To convert the concentrations of each benzimidazole into “DMB equivalents detected by LC/MS/MS,” the amount of each compound detected by LC/MS/MS was adjusted based on the yield of the extraction method and the EC_50_ value calculated by the *bluB* bioassay, and normalized to the values obtained for DMB.

## RESULTS

### THE CALCOFLUOR BRIGHT PHENOTYPE IS A RESPONSE TO DNA STRESS IN *S. meliloti*

**FIGURE 2:**
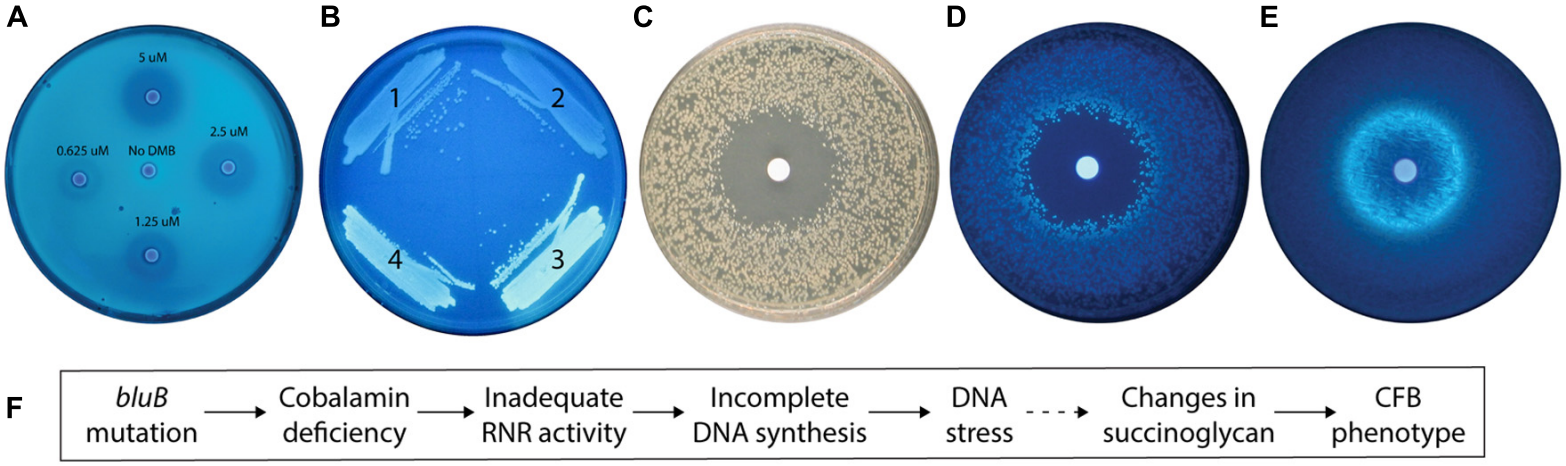
**The calcofluor (CF) phenotype of the *Sinorhizobium meliloti bluB* mutant is influenced by DMB availability and DNA stress. (A)** CF fluorescence of *S. meliloti bluB* on an LB CF plate. Filter disks containing DMB at the indicated concentrations were applied to the plate. **(B)** CF fluorescence phenotypes of *S. meliloti* strains (1) *bluB* mutant expressing *Escherichia coli nrdAB* on plasmid pMS03 ( Taga and Walker, 2010), (2) Wild type (WT) *S. meliloti* with empty vector pMS03, (3) *bluB* mutant with empty vector pMS03, and (4) *bluB* mutant expressing *E. coli metE* on plasmid pMS03 ( Taga and Walker, 2010). **(C)** Strain *Sm*NrdAB^+^ on an LB CF plate with a filter disk containing hydroxyurea, photographed under white light. **(D)** Same as **(C)** but photographed under UV light. **(E)** Fluorescence of WT *S. meliloti* on an LB CF plate with a filter disk containing nalidixic acid. Filter disks were applied to the plates at the time of inoculation. **(F)** Schematic description of the physiological link between the *bluB* mutation and the CF fluorescence phenotype. The dashed line indicates that the mechanism is unknown.

We previously found that the CFB phenotype of the *S. meliloti bluB* mutant can be rescued by the addition of DMB, which is used to produce cobalamin (Campbell et al., 2006; Taga et al., 2007). Bioassays, such as the bacterial assay that enabled the first purification of cobalamin (Rickes et al., 1948), can provide inexpensive, high-throughout, and highly sensitive means to detect molecules of interest (Kelleher and Broin, 1991). To investigate the possibility that this phenotype could be used to detect DMB, we first used a filter disk assay to examine the effect of DMB concentration on the CFB phenotype of a *bluB* mutant. This showed that the size and intensity of the CF-dim zone surrounding the DMB-soaked disks were positively correlated with the DMB concentration, suggesting that the CF phenotype could be used for quantification of DMB (**Figure 2A**).

To determine what other factors could influence the CF phenotype; we next characterized the physiological basis of the CFB phenotype of the *bluB* mutant. Our observation that cobalamin deficiency in *S. meliloti* results in a CFB phenotype suggests that one or more enzymes that use cobalamin as a cofactor can influence the amount or structure of succinoglycan. Two of the three known cobalamin-dependent enzymes in *S. meliloti*, methionine synthase and methylmalonyl-CoA mutase, were previously shown not to significantly affect the CF phenotype of *S. meliloti* and thus cannot explain the CFB phenotype of the *bluB* mutant (Campbell et al., 2006). Therefore, we hypothesized that the CFB phenotype could be a result of reduced activity of the Class II RNR encoded by *nrdJ*, a cobalamin-dependent enzyme that catalyzes the synthesis of deoxyribonucleotides (dNTPs; Cowles and Evans, 1968; Lawrence and Stubbe, 1998). To test this possibility, we expressed the *E. coli* cobalamin-independent (Class I) RNR encoded by *nrdA* and *nrdB* (Carlson et al., 1984) on a plasmid in a *bluB* mutant background and found that the strain has a dim phenotype on CF plates (**Figure 2B**). To further examine the link between the CFB phenotype and RNR activity, we used the *Sm*NrdAB^+^ strain (Δ*nrdJ* with the plasmid expressing *E. coli nrdAB*; Taga and Walker, 2010). The effect of hydroxyurea, a specific inhibitor of Class I RNRs (Elford, 1968), was tested by growing *Sm*NrdAB^+^ on an agar plate with a hydroxyurea-soaked filter disk. A zone of inhibition was formed surrounding the disk, indicating loss of viability due to inadequate RNR activity (**Figure 2C**). In addition, a CFB phenotype is evident in the colonies surrounding the zone of inhibition (**Figure 2D**). Together, these results indicate that the CFB phenotype can be induced by a reduction in RNR activity, either by direct inactivation by hydroxyurea treatment of the *Sm*NrdAB^+^ strain, or by cobalamin depletion such as in the *bluB* mutant.

Because inactivation of RNR results in a depletion of dNTP pools and replication fork arrest in *E. coli* (Guarino et al., 2007), we hypothesized that the CFB phenotype in the *bluB* mutant is a response to DNA stress. To test this possibility, we exposed WT *S. meliloti* to nalidixic acid, a DNA gyrase inhibitor that induces DNA damage (Pommier et al., 2010), and found that it induced a CFB phenotype adjacent to the zone of inhibition in the filter disk assay (**Figure 2E**). Treatment with four other DNA damaging agents resulted in a similar phenotype, suggesting that the CFB phenotype is induced in response to DNA damage (**Table 1**). To determine the range of compounds that induce the CFB phenotype, WT *S. meliloti* and the *Sm*NrdAB^+^ strain were analyzed using the filter disk assay with a variety of toxic agents. Of the 21 other compounds that induced a zone of inhibition, only three antibiotics, and SDS induced the CFB phenotype in WT *S. meliloti* (**Table 1**). Three other compounds, all of which cause intracellular oxidative damage, induced the CFB phenotype only in *Sm*NrdAB^+^, consistent with the previously observed sensitivity of this strain to reactive oxygen species (Taga and Walker, 2010). These results explain the physiological link between *bluB* and the CFB phenotype, as summarized in **Figure 2F**.

**Table 1:** Compounds tested for induction of CF bright phenotype in *Sinorhizobium meliloti*.

	Agents that induce a CFB ring in Rm1021, *Sm*NrdJ^+^, and *Sm*NrdAB^+^	Agents that do not induce a CFB ring	Agents that induce a CFB ring only in *Sm*NrdAB^+^
DNA damaging agents	Nalidixic acid, mitomycin C, cisplatin, MMS, 4NQO		
Antibiotics	Gentamycin, tetracycline	Chloramphenicol, spectinomycin, kanamycin	
Organic compounds		Methanol, butanol	
Metals and chelators		Cobalt, iron, EDTA, EGTA	
Acid/base		NaOH, formic acid	
Agents that cause membrane stress	SDS	Tween, triton, DMSO	
ROS and ROS-generating compounds			Hydroxyurea, hydrogen peroxide, menadione, cumene hydroperoxide

### CALCOFLUOR FLUORESCENCE PHENOTYPE OF *S. meliloti bluB* IN LIQUID MEDIA

**FIGURE 3:**
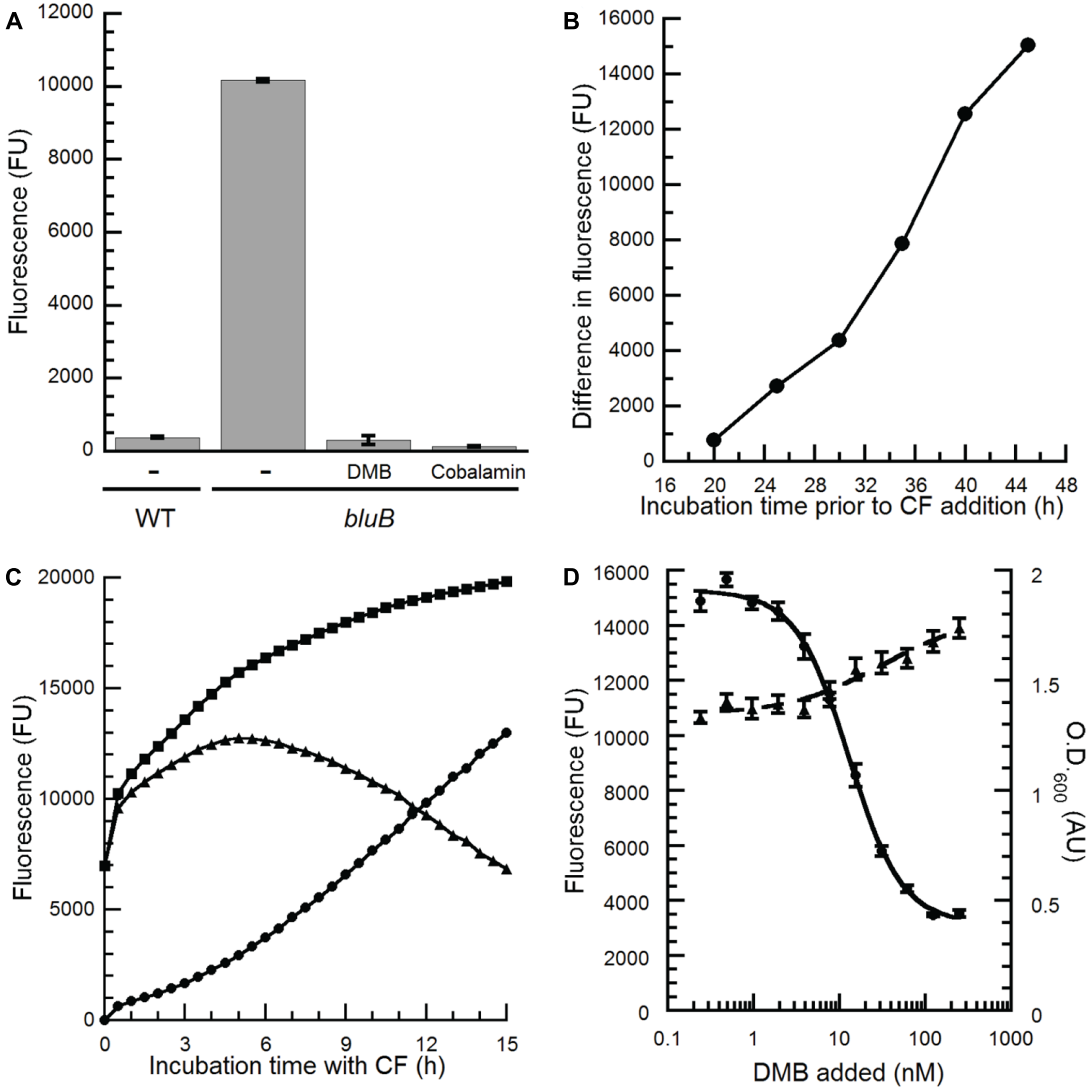
**Optimization of the *S. meliloti bluB* CF bioassay to quantify benzimidazoles. (A)** CF fluorescence of liquid cultures of WT *S. meliloti*, an *S. meliloti bluB* mutant (*bluB*), or *S. meliloti bluB* grown in the absence (-) or presence of 0.5 μM DMB or 1 μM cobalamin. Error bars represent the SE of three independent experiments. **(B)** Development of the CFB phenotype as a function of growth. *S. meliloti bluB* was grown in the presence and absence of 0.5 μM DMB. At the indicated time points, aliquots were removed and incubated with CF for 5 h. The difference in the CF fluorescence of cultures with and without DMB is plotted. **(C)** Development of the CFB phenotype as a function of incubation time with CF. After growth of *S. meliloti bluB* with (circles) and without (squares) DMB for 48 h, CF was added and fluorescence was measured at the indicated time points. Triangles represent the difference in fluorescence between the two cultures. **(D)** CF fluorescence (left axis, circles) and optical density (O.D._600_; right axis, triangles) is shown for the *S. meliloti bluB* mutant incubated in a 96-well plate with the indicated concentrations of DMB for 48 h followed by addition of CF for 5 h. Error bars represent the SE of six independent experiments. Fluorescence is presented in arbitrary units (FU).

The apparent dose-dependent relationship between the concentration of DMB and the CF phenotype on plates (**Figure 2A**) suggests that the CF phenotype of the *bluB* mutant could be used for the quantitative detection of DMB. To investigate whether the CFB response can be adapted for liquid media in a 96-well plate format, WT *S. meliloti* and a *bluB* mutant were cultured for 45 h in a 96-well plate, mixed with CF, and the fluorescence was measured in a multiwell plate reader. As on solid media, the *bluB* mutant showed significantly higher fluorescence than WT *S. meliloti*, with a 27-fold difference in fluorescence between the two strains (**Figure 3A**). Culturing the *bluB* mutant in the presence of DMB or cobalamin resulted in a low level of fluorescence comparable to that of WT *S. meliloti*, consistent with the phenotype on agar plates (**Figure 3A**; Campbell et al., 2006). A greater separation of the dim and bright phenotypes was observed as the bacteria were cultured for longer periods prior to the addition of CF (**Figure 3B**). Further optimization showed that the greatest difference in fluorescence was achieved when the cultures were incubated with CF for 5 h (**Figure 3C**). Based on these observations, the remaining CF fluorescence measurements were performed by incubating cultures with CF for 5 h following at least 55 h of growth in the 96-well plates.

**FIGURE 4:**
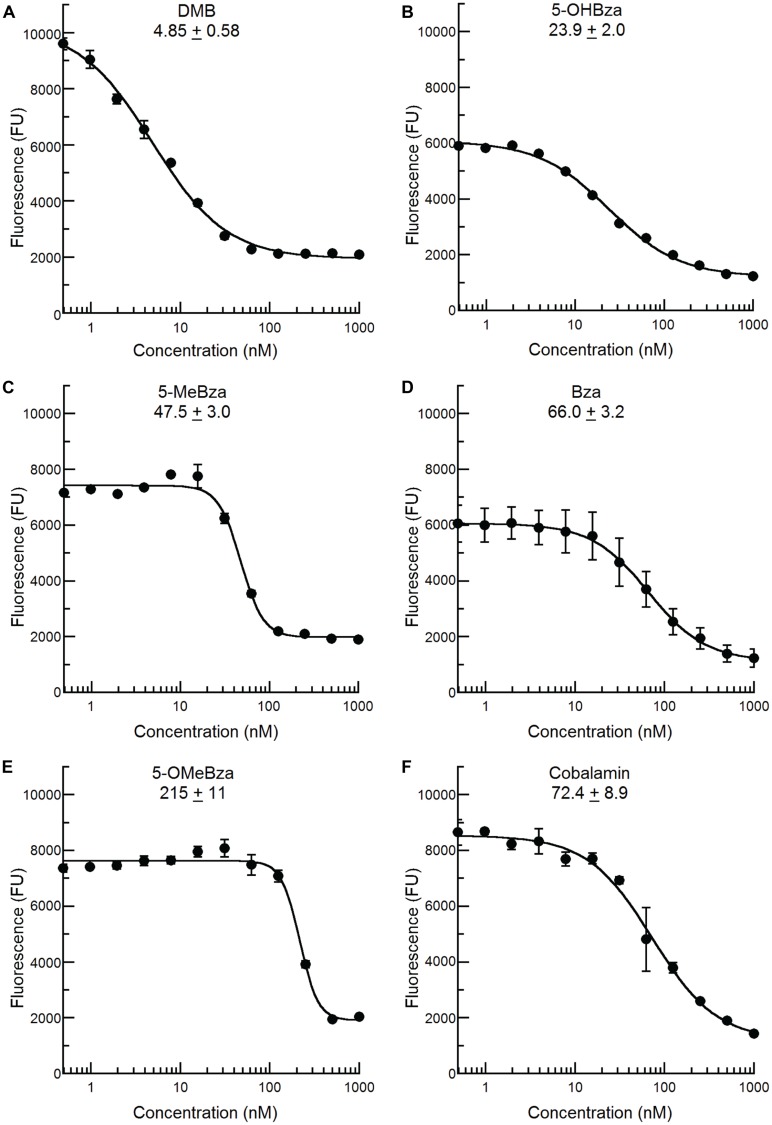
**Calcofluor response of *S. meliloti bluB* to other benzimidazoles and cobalamin.** The CF fluorescence of cultures grown with the indicated concentrations of **(A)** DMB, **(B)** 5-OHBza, **(C)** 5-MeBza, **(D)** Bza, **(E)** 5-OMeBza, and **(F)** cobalamin was assayed as shown in **Figure 3D**. The EC_50_ of each compound and curve-fit error are indicated on each graph. Error bars represent the SE of three independent experiments.

To determine whether the CFB phenotype showed a reproducible, dose-dependent relationship with DMB concentration, cultures of the *S. meliloti bluB* mutant were grown with DMB at concentrations ranging from 0.24 to 250 nM and incubated with CF. The results in **Figure 3D** show a relationship between fluorescence, and to a lesser extent the final O.D._600_ of the cultures, with the concentration of DMB added. The linear range of the curve was between 4 and 40 nM, and the concentration of DMB that resulted in half maximal fluorescence (EC_50_) was calculated to be 13 nM. The average and SD of the EC_50_ values were 7.37 and 5.59 nM, respectively, in 26 independent experiments. Replicates performed in a single experiment gave results with lower variation (**Figures 3D** and **4A**), demonstrating that this method can be used to detect DMB in unknown samples and quantify DMB concentrations if DMB standards are measured in the same experiment.

### THE CFB PHENOTYPE OF THE *S. meliloti bluB* MUTANT IS RESCUED IN A DOSE-DEPENDENT MANNER BY OTHER COBAMIDE-ASSOCIATED BENZIMIDAZOLES

We recently reported that an *S. meliloti bluB* mutant can incorporate a variety of benzimidazoles, including all of those in this study, into cobamides by guided biosynthesis, and that nearly all of these cobamides can support growth (Crofts et al., 2013; Hazra et al., 2013). In contrast, cobamides containing purines or phenolics do not support *S. meliloti* growth (Crofts et al., 2013). We therefore reasoned that the *bluB* bioassay could also be used to detect benzimidazoles other than DMB, and would not be subject to interference by cellular metabolites such as adenine. To test this possibility, the *bluB* bioassay was used to examine the CF response to the four other benzimidazoles shown in **Figure 1B**. All of these compounds were also able to rescue the fluorescence phenotype of the *bluB* mutant in a dose-dependent manner, though their EC_50_ values were 5- to 45-fold higher than for DMB (**Figure 4**). The addition of cobalamin similarly rescued the CFB phenotype, with an EC_50_ value 14-fold higher than for DMB (**Figure 4F**). These results demonstrate that this CF-based method can be used as a bioassay for detecting cobalamin and several benzimidazoles.

### DETECTION OF BENZIMIDAZOLES IN ENVIRONMENTAL SAMPLES

**FIGURE 5:**
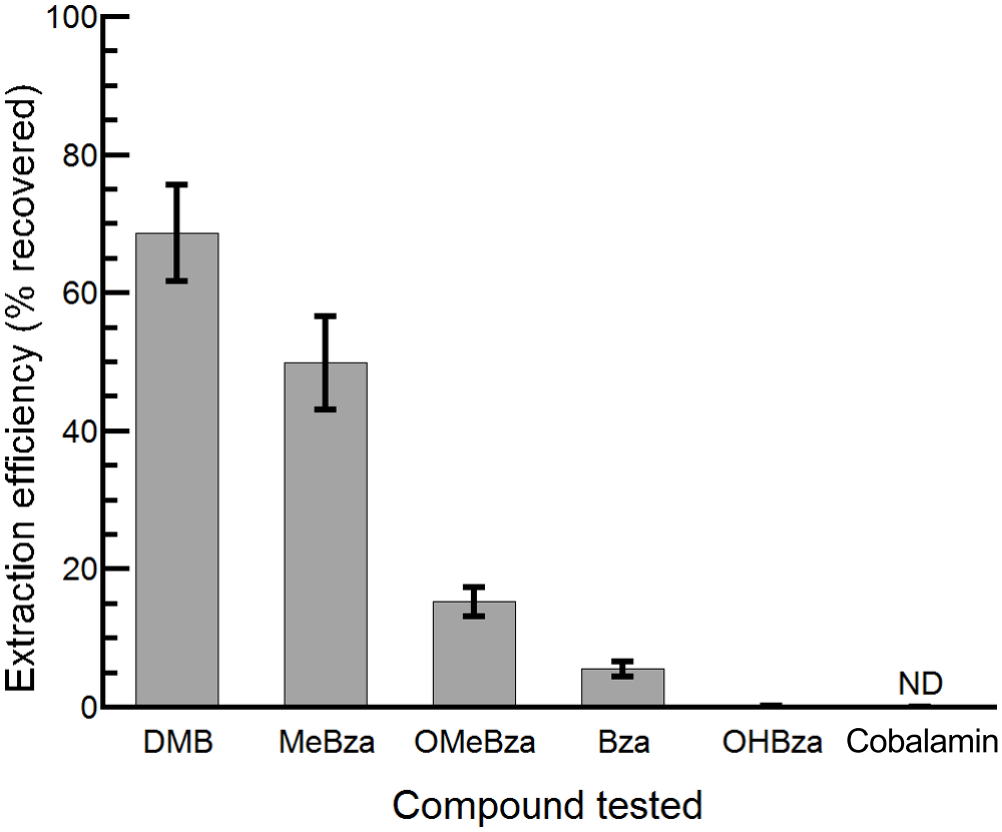
**Efficiency of extraction of benzimidazoles and cobalamin.** The percent recovery following extraction and purification of the indicated compounds is shown. Bars represent the average of three replicates, and error bars represent the SE. ND, not detected, limit of detection was 5 pmol.

The *bluB* bioassay was used to measure the concentrations of benzimidazoles present in environmental samples. Because environmental samples may contain compounds such as DNA damaging agents or cobamides that could interfere with the CFB response to benzimidazoles, and because the concentrations of benzimidazoles could be below our detection limit, we first developed a method to purify benzimidazoles from complex mixtures and concentrate them by up to 3000-fold. This method consists of a liquid–liquid extraction with ethyl acetate followed by C18 solid phase extraction (see Materials and Methods for details). The recovery of each benzimidazole varied following this purification method, with the highest yield observed for DMB and the lowest for 5-OHBza; **Figure 5**). Importantly, cobalamin could not be detected after this procedure, indicating that the bioassay results would not be influenced by cobalamin (or other corrinoids) that may have been present prior to purification (**Figure 5**).

To test whether the CF bioassay can quantify benzimidazoles present in complex mixtures, we performed the purification and bioassay with serial dilutions of spent media from cultures of WT *S. meliloti*, the *bluB* mutant, and two *bluB* mutant strains containing plasmids expressing *bluB* at different levels (Campbell et al., 2006; Yu et al., 2012). Strains containing the plasmid pMS03-*bluB* produce DMB at a high level such that when grown on solid media adjacent to a *bluB* mutant, they can induce a CF-dim phenotype in neighboring colonies (Yu et al., 2012). The plasmid pJL1031 does not have this ability, suggesting that strains containing this plasmid produce DMB at lower levels (Campbell et al., 2006). Consistent with these phenotypes, no DMB was detected in any of the samples except the strain containing pMS03-*bluB*. The concentration of DMB in this sample was found to be 400 nM based on dilutions whose CF fluorescence fell within the linear range of the DMB standard curve (**Table 2**). These results indicate that the CF bioassay can be used to measure DMB in biological samples.

**Table 2:** Benzimidazole levels in environmental samples as determined by the *S. meliloti* calcofluor bioassay and LC/MS/MS.

Sample	DMB equivalents detected by bioassay^a^	DMB equivalents detected by LC/MS/MS^b^	Benzimidazoles detected by LC/MS/MS				
			DMB	MeBza	OMeBza	Bza	OHBza
**Cell free supernatants**						
Wild type (nM)	NDB^c^						
*bluB* (nM)	NDB						
pMSO3 (nM)	398 ± 172						
pJl1031 (nM)	NDB						
**Laboratory agars**
Technical (pmol/g)	2.78 ± 1.16	1.81	0.242	0.286	0.231	2.48	6.67
Noble (pmol/g)	2.48 ± 1.66	0.615	0.307	0.370	0.347	0.974	0.942
Plant cell culture tested (pmol/g)	0.950 ± 0.458	0.0944	NQ^d^	0.196	NQ	1.01	NQ
Agarose (pmol/g)	1.84 ± 1.52	0.931	0.845	0.300	0.339	0.653	NQ
Yeast extract (pmol/g)	26.0 ± 1.21	8.28	6.89	7.42	1.84	4.99	1.10
**Host-associated samples**
Rumen 1 (pM)	262 ± 76.1	600	490	ND^e^	NQ	1500	ND
Rumen 2 (pM)	367 ± 33.9	654	562	ND	ND	1250	ND
Termite (pmol/g)	0.851 ± 0.255	0.240	0.181	0.259	NQ	0.442	ND
**Environmental samples**
Eucalyptus grove soil (pmol/g)	0.694 ± 0.291	0.0355	NQ	ND	ND	0.483	ND
Creek bank soil (pmol/g)	0.531 ± 0.361	0.0145	NQ	ND	NQ	0.197	ND
Creek water (pM)	7.36 ± 3.35	0.357	NQ	ND	NQ	4.86	ND
Winogradsky column water (pM)	104 ± 35.5	28.1	23.9	24.5	ND	22.6	ND

*^a^The total benzimidazole content is expressed as “DMB equivalents” because the bioassay does not differentiate DMB from other benzimidazoles. Average and SE of three independent biological replicates are shown.*

*^b^Expected bioassay equivalents, see Section “Materials and Methods” for the calculation method.*

*^c^NDB not detectable by bioassay, detection limit of 244 pM.*

*^d^NQ not quantifiable: less than 50 fmol. Compounds were detectable but too dilute for accurate quantification.*

*^e^ND not detected, compounds were below the detection limit of 20 fmol.*

It was previously reported that DMB is a contaminant of standard laboratory agar and that noble agar has less DMB contamination (Anderson et al., 2008). To measure the concentration of DMB in laboratory agars, we carried out the benzimidazole purification and CF bioassay on three grades of agar as well as agarose. Because the bioassay can detect multiple benzimidazoles, and because the purification yield and CF response to each benzimidazole varies, the results of the bioassay are reported as “DMB equivalents” which is calculated based on the extraction efficiency and the fluorescence response for DMB (see Materials and Methods). The bioassay detected benzimidazoles at levels equivalent to 1-3 pmol of DMB per gram of agar, and the levels did not correlate with the grade of agar (**Table 2**). We also measured the benzimidazoles present in commercial yeast extract by the same method. Surprisingly, the bioassay showed DMB equivalents 10 times higher than in the agar samples, despite the fact that yeasts are not known to synthesize benzimidazoles or cobamides.

The gastrointestinal tracts of several animals have been found to contain high concentrations of corrinoids. Bovine rumen, ovine tissue, wood-feeding insects, and the feces of humans, cows, and pigs have concentrations of corrinoids up to 2 μg per gram of sample, though the concentrations of free benzimidazoles in these environments have not been reported (Brown et al., 1955; Wakayama et al., 1984; Kelly et al., 2006; Allen and Stabler, 2008; Girard et al., 2009). We detected approximately 300 pM DMB equivalents in each of two bovine rumen fluid samples, while in termite fecal pellets ∼0.9 pmol/g was detected (**Table 2**). Environmental samples not associated with an animal host, including two soil samples and one creek water sample, contained DMB equivalents similar to termite feces (0.5–0.7 pmol/g and 7 pM, respectively; **Table 2**). In addition, approximately 100 pM DMB equivalents were measured in a laboratory Winogradsky column (**Table 2**).

To quantify the individual benzimidazoles present in each sample, we used a recently developed LC/MS/MS method that can detect and quantify the five benzimidazoles shown in **Figure 1B** (Men et al., 2014b). This method quantifies molecules based on specific retention times and unique fragmentation patterns by comparison to authentic standards. LC/MS/MS analysis indicated that nearly all of the samples contained multiple benzimidazoles in different concentrations. The total concentrations of benzimidazoles measured by LC/MS/MS were 2- to 10-fold lower than those calculated by the *bluB* bioassay in most cases, suggesting that additional benzimidazoles not included in our LC/MS/MS detection method could be present (**Table 2**). The benzimidazoles detected in our samples are likely not a result of abiotic degradation of cobamides during the purification process because an analysis of the cobamide content of some of the samples showed that cobamides corresponding to each benzimidazole were not present (data not shown). However, other products may lead to an overestimation of benzimidazole content in the bioassay, as discussed below. In sum, these results suggest that different combinations and concentrations of benzimidazoles are present in a variety of environments.

## DISCUSSION

Cobamides are important cofactors for diverse metabolic processes in the majority of prokaryotes as well as in animals and protists (Roth et al., 1996). Emerging work has shown that both cobamides and lower ligands are shared among microbes that reside in complex communities (Yan et al., 2012; Yi et al., 2012; Degnan et al., 2014; Men et al., 2014b). Guided biosynthesis and cobamide remodeling, two activities that require a free lower ligand base, have been observed in pure bacterial cultures, defined co-cultures, and communities, when amended with a lower ligand base (Gray and Escalante-Semerena, 2009; Yan et al., 2012; Yi et al., 2012; Men et al., 2014a). However, it is not known whether these activities can occur in environmental settings because the availability of free lower ligand bases has not been examined. Here, we have investigated the availability of free benzimidazoles in environmental samples. This work shows for the first time both the diversity and potential ubiquity of benzimidazoles in the environment.

We were surprised to find that benzimidazoles could be detected in all of the samples tested (except most of the *S. meliloti* supernatants). The presence of benzimidazoles in the soil samples was not surprising given the presence of Actinomycetes in soil, several of which are known to encode cobamide biosynthesis genes including *bluB* (Rodionov et al., 2003). Similarly, the bovine rumen and termite gut have high concentrations of cobamides, suggesting that benzimidazole biosynthesis occurs in these environments (Wakayama et al., 1984; Girard et al., 2009). The physiological relevance of the concentrations of benzimidazoles we observed remains unclear, as the highest levels (>100 pM) are slightly lower than those required to rescue the CFB phenotype of the *S. meliloti bluB* mutant (see **Figure 4**), and the minimum concentrations required for incorporation into cobamides in other bacteria remain untested. We previously observed that a 1,000-fold higher concentration of benzimidazoles is required to inhibit the growth of the cobamide-producing bacterium *Sporomusa ovata*, suggesting that the concentrations we observed would not drive guided biosynthesis in all bacteria (Mok and Taga, 2013). However, certain organisms that do not produce alternative lower ligands endogenously may be capable of salvaging benzimidazoles at these concentrations. Additionally, microenvironments within planktonic communities or biofilms could contain higher local concentrations of benzimidazoles that may affect nearby cells.

The diversity of benzimidazoles we observed may be underestimated, as our LC/MS/MS method detects only five of the six known cobamide-associated benzimidazoles (Renz, 1999), and other benzimidazoles that have not yet been observed in cobamides may be biologically relevant. It is possible that other benzimidazoles are present that were not detected by LC/MS/MS since the total benzimidazole concentration estimated by LC/MS/MS was lower than the concentration measured by the bioassay in the majority of samples. Conversely, the benzimidazole levels measured in the bioassay may have been overestimated because compounds other than benzimidazoles that cause a reduction in CF fluorescence could be present in the samples, or the bioassay may have collectively detected trace amounts of benzimidazoles that, when measured individually, fell below the quantifiable range of the LC/MS/MS method. Finally, we cannot formally rule out the possibility that some of the compounds measured as benzimidazoles by LC/MS/MS could instead be other compounds present in the samples that have chemical properties very similar to a benzimidazole being detected, including the same retention time, m/z (to within ∼0.1 amu), and fragmented product ion m/z values.

Our detection of DMB (and other benzimidazoles) in laboratory agars is consistent with a previous study that reported genetic evidence that DMB activity is present in standard laboratory agar at levels sufficient to promote guided biosynthesis in *Salmonella enterica* (Anderson et al., 2008). However, it is unclear why we detected similar concentrations of benzimidazoles in both technical and noble agars, though this discrepancy could be explained by a strong response to Bza and 5-OHBza, which were found at higher levels in technical agar, in *S. enterica*. Given that agar is derived from algae, it is likely that the benzimidazoles present in agar originate from cobamide-producing bacteria associated with the algal cells (Croft et al., 2005; Helliwell et al., 2011; Kazamia et al., 2012).

We were particularly surprised to detect a substantial amount of benzimidazoles in yeast extract, since fungi are reported not to synthesize cobamides. The presence of these compounds points to possible cobamide-independent roles for benzimidazoles. One example of a naturally occurring benzimidazole that is not associated with a cobamide is kealiiquinone, a compound extracted from a sponge of the *Leucetta* genus, which indicates that benzimidazole synthesis is not limited to prokaryotes (Akee et al., 1990; Alamgir et al., 2007). Other benzimidazoles have been found to have biological activities not clearly associated with cobamide synthesis, such as inhibition of biofilm formation (Sambanthamoorthy et al., 2011). Alternatively, DMB may be formed non-enzymatically from flavins during the production of yeast extract, though the production of benzimidazoles other than DMB in this manner would require additional steps (Maggio-Hall et al., 2003). Bacterial contamination during industrial preparation is another possible source of benzimidazoles in yeast extract. Regardless of the source, these compounds were not previously recognized as components of laboratory yeast extract, and their presence in bacterial growth media could influence cobamide-dependent biological processes. For example, the presence of benzimidazoles in yeast extract and agar likely explains why the *S. meliloti bluB* mutant, but not the *cobU* mutant, can grow without cobalamin supplementation in LB liquid medium and on minimal agar plates. The *bluB* mutant, which is unable to synthesize DMB, is apparently partially rescued by benzimidazoles in the media, while the *cobU* mutant, which is unable to activate lower ligand bases for attachment, cannot use the benzimidazoles available in the media (Campbell et al., 2006; Crofts et al., 2013). The CFB phenotype of the *bluB* mutant, which is utilized in the bioassay described here, therefore indicates a partial rescue of the DMB auxotrophy by benzimidazoles in the media.

Here, we also present evidence that the CFB phenotype of the *bluB* mutant is the result of DNA stress due to inadequate activity of the cobalamin-dependent RNR. Although DNA stress was not previously known to elicit a CFB response, the CFB phenotype has been observed in response to several other types of stress including low nutrient conditions and oxidative stress (Wells and Long, 2002). This response is likely due to the ability of EPSs including succinoglycan to promote biofilm formation and protect against toxins including reactive oxygen species produced by the plant host during nodule invasion (Fujishige et al., 2006; Nogales et al., 2006; Rinaudi et al., 2006; Soto et al., 2006; Lehman and Long, 2013). The absence of a mucoid colony phenotype in the *bluB* mutant suggests that the CFB phenotype is due, at least in part, to a structural alteration in succinoglycan, rather than overproduction. We do not yet know the structure of the altered succinoglycan produced in response to DNA stress, or the benefit, if any, this altered EPS provides.

We have used two independent methods, a newly developed bioassay and an LC/MS/MS method, to detect benzimidazoles in a variety of environments. The prevalence of these compounds in both aerobic (such as soil and creek water) and anaerobic environments (such as rumen) is also interesting from a biochemical perspective because their biosynthetic pathways are unknown, with the exception of the aerobic biosynthesis of DMB which is catalyzed by BluB (Campbell et al., 2006; Taga et al., 2007). Because it can be performed in a high-throughput manner, the bioassay described here could be used as a tool to screen for benzimidazole production to aid in the identification of yet undiscovered benzimidazole biosynthetic genes such as the elusive anaerobic pathway to DMB (Renz, 1999). The identification of benzimidazoles in diverse environments and their roles in cobamide biosynthesis, salvaging, and remodeling reinforces the importance of identifying these pathways.

## Conflict of Interest Statement

The authors declare that the research was conducted in the absence of any commercial or financial relationships that could be construed as a potential conflict of interest.
